# Evaluation of a Motivational Interviewing-Based Training Programme in Vaccination Counselling for Family Physicians: A Pre–Post Study in Romania

**DOI:** 10.3390/vaccines14090782

**Published:** 2026-09-07

**Authors:** Roxana Surugiu, Virginia-Maria Rădulescu, Anca Deleanu, Mirela Mustață, Dana Fărcășanu, Iulia Vișinescu, Alexandra-Aurora Dumitra, Gheorghe Gindrovel Dumitra

**Affiliations:** 1 Biochemistry Department, University of Medicine and Pharmacy Craiova, 200349 Craiova, Romania; 2 Department of Medical Informatics and Biostatistics, Faculty of Medicine, University of Medicine and Pharmacy of Craiova, 200349 Craiova, Romania; 3 Immunisation Working Group, National Society of Family Medicine, 024076 Bucharest, Romania; anca_deleanu2001@yahoo.com (A.D.); gindrovel.dumitra@umfcv.ro (G.G.D.); 4 Centre for Health Policies and Services, 011066 Bucharest, Romania; mirelamustata@gmail.com (M.M.); iuliavisinescu@gmail.com (I.V.); 5 Doctoral School, University of Medicine and Pharmacy Craiova, 200349 Craiova, Romania; 6 Family Medicine Discipline, University of Medicine and Pharmacy Craiova, 200349 Craiova, Romania

**Keywords:** motivational interviewing, physician training, vaccine hesitancy, family medicine, continuing medical education, communication skills, Romania

## Abstract

**Background/Objectives**: Motivational interviewing (MI) is an established approach to addressing vaccine hesitancy, yet immediate changes following dedicated training for family physicians remain insufficiently documented in Romania. This study evaluated pre–post changes in physicians’ MI knowledge, self-reported communication behaviour, and self-perceived ability to counsel patients about vaccination, together with patient-level change following consultations delivered by trained physicians. **Methods**: This uncontrolled pre–post study used a voluntary, self-selected non-probability sample of 28 family physicians recruited through announcements distributed via county family medicine associations; participants completed paired assessments immediately before and after structured MI-based training. The three main physician-level outcomes were MI knowledge (MISI), corrected self-reported MI communication behaviour (MIBI), and self-perceived MI ability (SPMI). The linked component comprised 151 patients nested within 15 physicians. Patient changes were averaged within physician, and the physician-level mean patient composite change was used as the principal outcome of this component. Paired physician changes and physician-aggregated patient change were assessed using Wilcoxon tests, rank-biserial effect sizes, and bootstrap 95% confidence intervals (CIs), with Holm adjustment across the three physician outcomes. **Results**: Mean MISI increased by 39.80 percentage points (95% CI: 29.59–50.51), though the limited internal consistency of this seven-item composite requires item-level interpretation, and corrected MIBI by 0.44 points (95% CI: 0.20–0.68; r_rb_ = 0.75) and SPMI by 1.22 points (95% CI: 0.89–1.61; r_rb_ = 0.99); all Holm-adjusted *p*-values were <0.001. Across the 15 linked physicians, the mean patient composite change was 0.55 points (95% CI: 0.38–0.75; r_rb_ = 1.00; *p* < 0.001). No consistent association was observed between physician-level competency changes and mean patient change. **Conclusions**: Immediate post-training physician scores and post-consultation patient composite scores were higher. Controlled studies incorporating observed consultations and follow-up are required before causal or sustained effects can be inferred.

## 1. Introduction

Vaccine hesitancy (VH) is recognised as one of the main threats to public health [1]. In 2019, prior to the COVID-19 pandemic, the World Health Organisation (WHO) classified vaccine hesitancy among the top 10 global health threats [2]. The VH phenomenon has also been visible in Romania over the past 18 years and has led to the failure of the vaccination program aimed at preventing diseases caused by the human papillomavirus (HPV) [3], a decline in vaccination coverage in children for the vaccines included in the National Immunization Program (NIP) [4], and has fostered a widespread public misconception regarding the effectiveness of adult vaccinations [1,5,6]. Romania experienced persistently low COVID-19 vaccination uptake throughout the pandemic, reflecting multiple interacting determinants of vaccine acceptance [7,8,9,10]. Moreover, the recent legislative changes regarding the reimbursement of vaccines for at-risk population groups, including adults, have encountered low acceptance among the beneficiary population [11,12]. The case of HPV vaccination illustrates this disconnect, with 90% of physicians offering HPV vaccination services and 88.5% considering themselves well informed, yet attitudes—of both physicians and the public—are only moderately positive, which limits coverage. This directly supports the argument that the barrier is not only knowledge but also other causes, including physicians’ communication skills [12].

This phenomenon has posed particular challenges for family physicians in Romania as well as in many other European countries [12,13] that have not experienced the transition from mandatory vaccination to vaccination recommendation. These dynamics are important for the primary healthcare providers because, in Romania, family physicians’ practice is the main vaccination provider for both children and adults, and the medical staff working at this level is considered the most important source of trust for hesitant patients [14]. The quality of the physician–patient interaction thus becomes a central determinant of vaccination coverage, and the physician’s communication skills are at least as important as their medical knowledge [15,16,17]. Trust is consequential because a recommendation affects uptake only if the source is regarded as both competent and respectful of patient autonomy; conversely, mistrust can make even accurate information less persuasive [14,16,17]. Longitudinal evidence documenting a specific decline in trust in Romanian family physicians is not available, but the broader erosion of institutional and vaccine confidence during and after the COVID-19 period makes the quality of primary-care vaccination conversations especially relevant [7,8,9,10].

Numerous studies have shown that simply providing information sometimes corrects hesitancy but may even reinforce resistance when the insistence of health care workers is perceived as pressure [15,18,19]. By contrast, relational, patient-centred approaches that explore and resolve ambivalence while respecting the person’s autonomy are more effective. Motivational interviewing (MI) is a collaborative, goal-oriented counselling style within the broader family of behaviour-change interventions; it originated in clinical psychology but is not, in this context, psychotherapy. It seeks to elicit the patient’s own reasons for change through partnership, acceptance, compassion, and evocation, using skills such as open questions, affirmations, reflective listening, and summaries. MI has been used in brief healthcare encounters addressing substance use, treatment adherence, lifestyle change, and other preference-sensitive behaviours, and it has been specifically adapted for vaccination counselling [18,19]. It is the prototype of these relational approaches and has been specifically adapted for the immunisation context. Among the strongest evidence comes from the Canadian PromoVac study and derived programs, which demonstrated that professionals trained in MI achieve reductions in hesitancy and increases in vaccination coverage, with effects that persist over time [20,21]. Likewise, empathetic communication techniques through the empathetic refutational interview have yielded good results in Romania, increasing physicians’ self-confidence as well as patients’ vaccination acceptance [22,23].

The effectiveness of the intervention and its effect on patient behaviour are, however, conditioned by an often-unverified intermediate link—the extent to which professionals actually acquire and apply MI competencies following training. A programme may be well received yet not change the physician’s communication behaviour; conversely, real competency gains do not automatically guarantee changes at the patient level if structural barriers persist [17,24,25]. Rigorous evaluation of the training’s effect on the physician—knowledge, behaviours, and self-efficacy—is therefore essential for correctly interpreting patient-level results and for optimising programmes. Clinician competency assessment is not absent from the previous literature. Rather, existing educational evaluations remain heterogeneous in their content, assessment methods, and outcome selection. Much of the vaccination literature has prioritised patient hesitancy, intention, or uptake, whilst professional-training studies have often focused on acceptability or a single self-reported outcome. Fewer evaluations distinguish knowledge acquisition from self-reported communication behaviour and confidence, and fewer still examine whether physician-level change covaries with a proximal patient response [20,21,22,23,25]. Immediate competency assessment is therefore not merely a local replication: it helps locate where the proposed training → competency → patient-response pathway may strengthen or fail before sustained clinical effectiveness is claimed.

Although Romanian evidence remains limited [26], the principal gap is not geographical alone. It concerns the immediate and differentiated acquisition of MI-related knowledge, self-reported communication behaviour, and confidence, together with the exploratory link between physician-level change and proximal patient response. The present study therefore examined immediate pre–post changes across these three physician domains and evaluated, as a linked exploratory component, physician-aggregated patient change following vaccination counselling.

## 2. Materials and Methods

### 2.1. Study Design and Setting

We conducted a pre–post intervention study in which each physician served as their own control (measurements before and immediately after training). The study was carried out in Romania in 2025, in two counties (Iași and Dolj, with two sessions held in the cities of Iași and Craiova). The programme was jointly developed by the Centre for Health Policies and Services (CPSS) and the Vaccinology Group of the National Society of Family Medicine in Romania (SNMF), and was accredited with continuing medical education credits. The study had a two-level design comprising (i) a physician-level evaluation of immediate pre–post changes in motivational interviewing competencies, which constituted the main focus of the study, and (ii) a linked physician–patient component based on assessments completed before and after vaccination counselling delivered by the trained physicians in their own practices. Within the linked component, individual patient changes were aggregated within physicians, allowing the physician-level mean patient composite change to be evaluated whilst accounting for the clustering of patients within practices. The present study reports the physician-aggregated patient change and explores its association with changes in physician competencies.

### 2.2. Training Programme

Pneumococcal vaccination was selected as the clinical context because recent reimbursement policies have expanded access to vaccination for at-risk adults, while uptake remains limited, providing a relevant setting in which to develop communication skills for addressing vaccine hesitancy. The programme combined the updating of knowledge about pneumococcal disease and vaccination with an interactive MI-centred communication module: the principles and spirit of MI, reflective listening, open questions, evoking the patient’s own motivation, recognising and addressing resistance and counterarguments, and respecting autonomy. Training was delivered in person by trainers experienced in MI and vaccinology, using adult-specific teaching methods such as presentation, examples, role-play, small-group work, brainstorming, and feedback. The educational sequence comprised four connected components: (1) a clinical update on pneumococcal disease, vaccine indications, and the contemporary reimbursement context; (2) introduction to the relational spirit and principles of MI; (3) demonstration and practice of open questions, reflective listening, eliciting the patient’s own motivation, and responding to resistance without confrontation; and (4) application to vaccine-hesitancy scenarios through examples, role-play, small-group work, brainstorming, and trainer feedback. Both sites used this common programme framework and face-to-face format. A formal session-level fidelity checklist or independent observation of trainer delivery was not included; equivalence of delivery between the two sessions therefore cannot be demonstrated.

### 2.3. Participants

Participants were recruited through voluntary, self-selected non-probability sampling after an announcement distributed via the communication channels of the county family medicine associations in Iași and Dolj. Eligible participants were practising family physicians who owned an individual medical practice, held a vaccination-service contract with the relevant county public health directorate, attended the training, and provided linkable pre- and immediate post-training assessments. Physicians who did not meet the vaccination-service criterion or did not provide a paired assessment were ineligible for the paired analysis. Participation and completion of the instruments were voluntary. Initials were used solely to link pre- and post-training assessments and, where applicable, aggregated patient outcomes; the analytical dataset was subsequently coded and contained no direct identifiers. Participant numbers and the flow into the analysis are reported in Section 3.1.

An a priori sample-size calculation was performed using G*Power version 3.1.9.7 for a two-tailed paired-samples comparison. Based on previous evidence indicating the moderate effects of structured communication training among healthcare professionals [25], a standardised within-participant effect size of *d_z_* = 0.58 was assumed. With an alpha level of 0.05 and 80% statistical power, a minimum of 26 physicians with complete paired assessments was required. The planned sample was increased to 28 physicians to allow for incomplete or unmatched pre–post assessments. All 28 physicians completed both assessments, corresponding to a design power of approximately 84% under the assumed effect. The paired-samples t-test framework was used as a planning approximation for the intended paired pre–post comparisons; the non-parametric methods used for the final analyses are described in Section 2.6.

### 2.4. Assessment Instruments

Competencies were assessed using the Motivational Interviewing Skills in Immunisation (MISI) questionnaire [27], which measures three dimensions of MI competence in immunisation: knowledge, behaviour, and self-perceived confidence. We used the shortened Romanian-language version previously employed in this setting [23], with the same three subscales completed before and immediately after training: (i) the MI knowledge subscale (referred to here as MISI), comprising 7 items scored correct/incorrect (total 0–7 and percentage correct); (ii) the MI communication-behaviour subscale (MIBI), comprising 12 items scored from 1 to 6, with items 5 and 9 describing MI-inconsistent behaviours and reverse-scored so that higher composite scores consistently indicate more MI-aligned communication; and (iii) the self-perceived ability/confidence subscale (SPMI), comprising 6 items scored from 1 to 10. The first ten five-point Likert items in the physician form assessed general vaccination attitudes and were not included in the present competency outcomes. Thus, MISI, MIBI, and SPMI refer to the knowledge, self-reported communication-behaviour, and self-perceived confidence subscales, respectively, of the validated MISI questionnaire. The original instrument has published psychometric support [27]; reliability in the present sample was assessed separately because a shortened Romanian-language version was used. Published psychometric support for the original MISI instrument cannot be assumed to transfer unchanged to the shortened Romanian-language version used here. No validated adequacy cut-off has been established for the shortened Romanian MISI, corrected MIBI, or SPMI scores used here. Higher scores indicate more correct knowledge, more MI-aligned self-reported behaviour, or greater self-perceived ability, respectively; consequently, the outcomes were analysed as continuous scores and interpreted through the magnitude and direction of paired change rather than by classifying physicians as competent or non-competent.

The linked patient assessment was designed to capture a proximal response to the vaccination consultation rather than verified vaccine uptake. Patients completed the same brief form before and after counselling. This form comprised seven vaccination-attitude items and one vaccination-intention item, each rated from 1 to 7; two negatively oriented attitude items were reverse-scored so that higher values consistently represented more favourable attitudes or intention. The equally weighted composite was chosen to summarise the direction of immediate patient response whilst avoiding multiple underpowered item-level tests. Its scoring and physician-level aggregation are described in Section 2.6.

### 2.5. Ethics

The study was conducted in accordance with the Declaration of Helsinki and was approved by the Ethics Committee of the University of Medicine and Pharmacy of Craiova, Romania (Approval No. 118/10.02.2025). Participation was voluntary, and the data used for linking the pre- and post-assessments were coded before analysis.

### 2.6. Statistical Analysis

Item responses were checked against their permitted ranges before scoring. A response recorded as ‘n/r’ and one MIBI response outside the 1–6 scale were treated as missing. MISI was calculated as the sum of seven binary items (0–7) and expressed as percentage correct. MIBI items 5 and 9 were reverse-scored as 7 minus the observed response, and the corrected MIBI score was the mean of the available valid items. SPMI was the mean of six items scored from 1 to 10. No single outcome was prospectively designated as the sole primary outcome; MISI, corrected MIBI, and SPMI were therefore treated as a family of three main scale-level outcomes, with Holm adjustment used to control the family-wise error rate. Outcomes are summarised using mean ± standard deviation and median [interquartile range]. Mean paired changes and matched-pairs rank-biserial correlations are reported with percentile bootstrap 95% confidence intervals based on 10,000 paired resamples using a fixed seed (20260807). Paired changes were tested using two-sided Wilcoxon signed-rank tests and quantified using matched-pairs rank-biserial correlation (r_rb_). MISI item changes were assessed using exact McNemar tests with Holm adjustment across seven items. The two MI-inconsistent MIBI items were analysed on their original scales, with Holm adjustment across the two item-level comparisons. Centre-specific within-group Wilcoxon analyses were exploratory. Differences in individual change scores between Iași and Dolj were examined directly using Mann–Whitney U tests with between-group rank-biserial correlations and Holm adjustment across the three outcomes. Internal consistency was estimated using KR-20 for MISI and Cronbach’s alpha for MIBI and SPMI. Complete-case analyses for MIBI and SPMI were performed as sensitivity analyses. For the linked physician–patient component, the physician-level mean patient composite change was used as the principal summary outcome. The patient composite comprised seven vaccination-attitude items and vaccination intention, all scored from 1 to 7 and weighted equally; items 4 and 7 were reverse-scored as 8 minus the observed response. Pre- and post-consultation scores were calculated as the mean of the available valid components, and positive post-minus-pre values indicated change towards more favourable vaccination attitudes and intention. All included patient records contained at least seven valid components at both assessments. Individual patient changes were first averaged within physician, and the physician was retained as the independent unit of analysis to account for the clustering of patients within practices. The distribution of physician-level mean patient changes was summarised using the mean ± standard deviation and median [interquartile range]. The pooled mean change and its percentile-bootstrap 95% confidence interval were estimated using 10,000 physician-level resamples. Change relative to zero was examined using a two-sided one-sample Wilcoxon signed-rank test and quantified using the rank-biserial correlation. An analysis weighted by the number of patients contributing to each physician-level mean was performed as a sensitivity analysis. Associations between mean patient composite change and physician changes in MISI, corrected MIBI, and SPMI were examined separately within each centre using Spearman’s rho. Percentile-bootstrap 95% confidence intervals were obtained from 10,000 physician-level resamples, and Holm adjustment was applied across the six exploratory correlations. All tests were two-sided. Exact *p* values are reported to three decimal places, with *p* < 0.001 used for smaller values. The a priori sample-size calculation was performed using G*Power, version 3.1.9.7 (Heinrich Heine University Düsseldorf, Düsseldorf, Germany). Primary data were organised and coded in Microsoft Excel 2021 (Microsoft Corporation, Redmond, WA, USA), and statistical analyses were performed using IBM SPSS Statistics, version 26.0 (IBM Corp., Armonk, NY, USA).

Additional exploratory analyses were undertaken to characterise the pattern and relative magnitude of change without redefining the three main outcomes. To permit descriptive comparison across instruments with different metrics, each participant’s change was expressed as a percentage of the theoretical scale range (0–7 for MISI, 1–6 for corrected MIBI, and 1–10 for SPMI); mean normalised changes and paired between-outcome contrasts were estimated using 10,000 paired bootstrap resamples. The number of physicians with positive change in all three or in at least two domains was also summarised descriptively; no threshold for educationally meaningful change was prespecified. To clarify the MIBI result, the ten MI-aligned items and the two MI-inconsistent items were additionally summarised as separate exploratory domains; the latter were retained on their original scales, on which lower values indicate a more MI-consistent direction. Wilcoxon tests for these two MIBI domains were Holm-adjusted as a two-comparison family. The six SPMI items were examined separately with Holm adjustment across six comparisons. Immediate programme acceptability was described using five course-evaluation items rated from 1 to 5, with higher values indicating more favourable evaluations: perceived learning, anticipated influence on practice, organisation, scientific content, and lecturer quality.

## 3. Results

### 3.1. Participants and Main Scale-Level Outcomes

All 28 physicians with paired pre- and post-training assessments were included: 18 from Iași and 10 from Dolj. All were practising family physicians holding vaccination-service contracts. The assessment dataset was limited to centre, eligibility, linkage, and study-outcome variables; age, sex, years in practice, practice setting, and previous communication training were not available. The sample therefore cannot be demographically or professionally characterised beyond the stated eligibility criteria, and selection or effect heterogeneity across these unmeasured characteristics cannot be evaluated. One MIBI response was recorded as ‘n/r’ and one out-of-range MIBI response was treated as missing, and the corresponding scores were calculated from the remaining valid items. The MIBI complete-case sensitivity analysis therefore included 26 physicians. One physician had incomplete SPMI data, yielding 27 complete cases for the SPMI sensitivity analysis. MISI data were complete for all participants.

In the pooled sample, mean MISI scores increased from 29.08% ± 20.75% to 68.88% ± 23.02%, corresponding to a mean paired increase of 39.80 percentage points (bootstrap 95% CI: 29.59–50.51). The median paired increase was 28.57 percentage points [IQR: 14.29–57.14], and the matched-pairs rank-biserial correlation was 0.98 (bootstrap 95% CI: 0.91–1.00). The corresponding pre- and post-training medians were 28.57% [14.29–42.86%] and 71.43% [53.57–85.71%]. Corrected MIBI increased from 4.32 ± 0.63 to 4.76 ± 0.52, with a mean paired increase of 0.44 points (95% CI: 0.20–0.68), a median paired increase of 0.42 [0.21–0.69], and r_rb_ = 0.75 (95% CI: 0.42–0.99). The corresponding pre- and post-training medians were 4.46 [3.88–4.77] and 4.92 [4.42–5.17]. SPMI increased from 7.83 ± 1.33 to 9.05 ± 0.82, with a mean paired increase of 1.22 points (95% CI: 0.89–1.61), a median paired increase of 1.08 [0.50–1.38], and r_rb_ = 0.99 (95% CI: 0.97–1.00). The corresponding pre- and post-training medians were 8.00 [7.33–8.83] and 9.33 [8.75–9.67]. All three main scale-level outcomes remained statistically significant after Holm adjustment (all adjusted *p* < 0.001). Pooled and centre-specific estimates are summarised in Table 1. Because the pre-training KR-20 was 0.42, the composite change should not be read as a precise estimate of change in a single, highly coherent knowledge construct; the item-level pattern in Section 3.2 is central to its interpretation.

**Figure 1:**
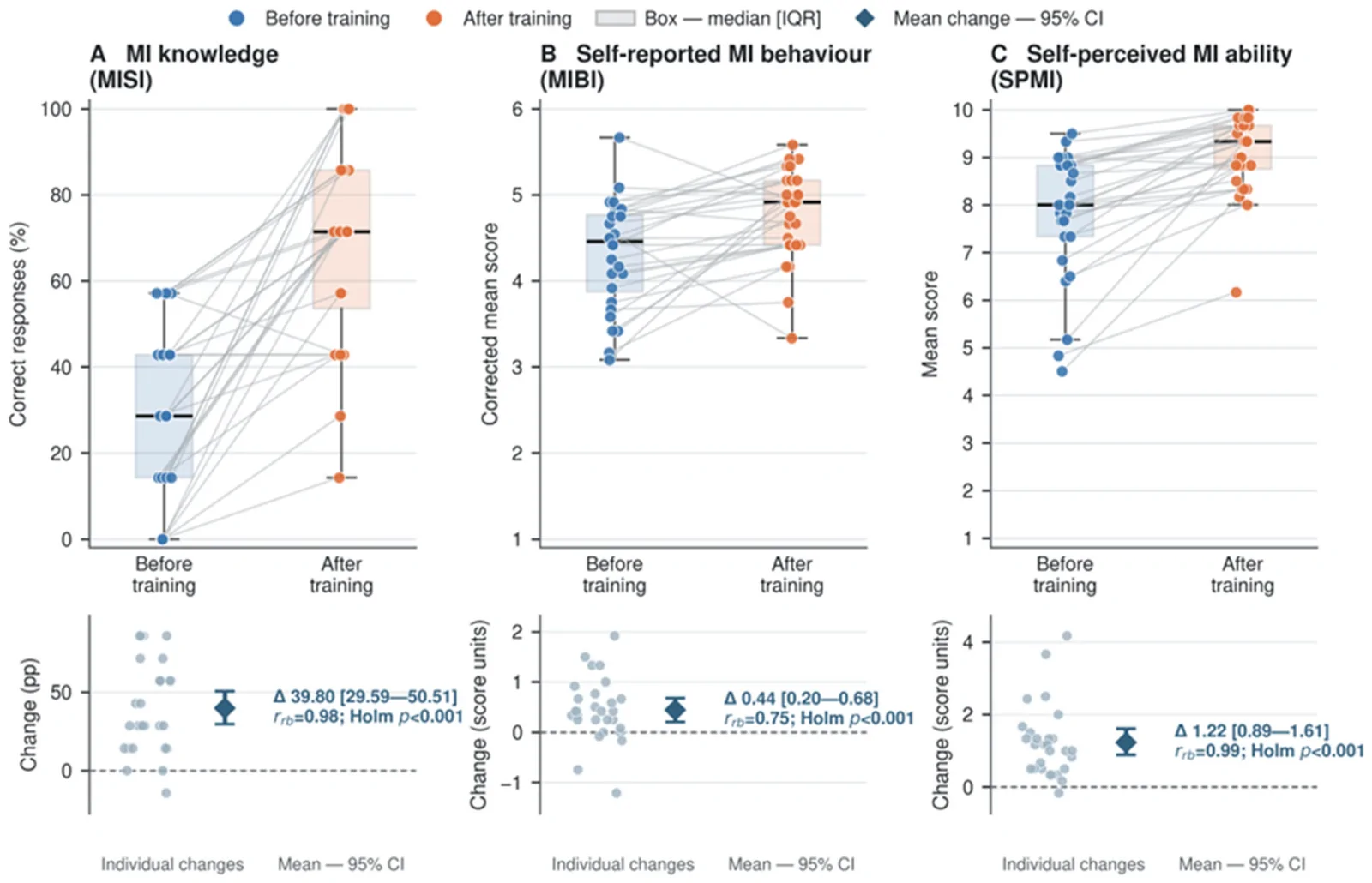
Paired pre–post physician outcomes in the pooled sample (*n* = 28): MI knowledge (MISI; (**A**)), corrected self-reported MI behaviour (MIBI; (**B**)), and self-perceived MI ability (SPMI; (**C**)). Points and connecting lines show individual observations; boxes show medians and interquartile ranges. Lower panels show paired changes, mean changes, and percentile-bootstrap 95% confidence intervals. Holm-adjusted *p* values and matched-pairs rank-biserial correlations (rrb) are reported.

Positive changes were observed for 25 physicians on MISI, 22 on corrected MIBI, and 27 on SPMI. MISI scores were unchanged for two physicians and decreased for one; corrected MIBI scores were unchanged for two and decreased for four; one physician reported a lower SPMI score, and none was unchanged. Paired individual trajectories are shown in Figure 1.

For descriptive comparison across the different instrument metrics, mean changes represented 39.80% of the theoretical scale range for MISI (bootstrap 95% CI: 29.59–50.51), 13.60% for SPMI (95% CI: 9.85–17.90), and 8.85% for corrected MIBI (95% CI: 4.07–13.57). In exploratory paired bootstrap contrasts, the normalised MISI change exceeded the corrected MIBI change by 30.95 percentage points (95% CI: 21.23–40.73) and the SPMI change by 26.20 percentage points (95% CI: 17.58–35.57). These cross-instrument comparisons were considered descriptive because the measures differed in response format, reliability, and potential ceiling effects. Eighteen of the 28 physicians (64.29%) showed positive changes in all three outcomes, and all 28 showed a positive change in at least two. This analysis describes directional consistency only, as no threshold for educationally meaningful improvement had been defined.

### 3.2. Centre-Specific, Item-Level, and Robustness Analyses

Within-centre analyses showed directionally consistent increases in MISI and SPMI in both Iași and Dolj, whilst corrected MIBI increased in both centres but was statistically supported only in Iași (Table 1). These separate within-centre tests were not interpreted as evidence of different training-related changes. Direct exploratory comparisons of individual change scores between Iași and Dolj yielded Mann–Whitney U = 104.50 for MISI (raw *p* = 0.499; Holm-adjusted *p* = 0.499; between-group r_rb_ = 0.16), U = 115.00 for corrected MIBI (raw *p* = 0.239; adjusted *p* = 0.478; r_rb_ = 0.28), and U = 54.00 for SPMI (raw *p* = 0.088; adjusted *p* = 0.265; r_rb_ = −0.40). Thus, no between-centre difference was statistically supported, although the small centre-specific samples precluded conclusions of equivalence. In Iași, improvements were observed for 15 of 18 physicians on MISI, 14 of 18 on MIBI, and 17 of 18 on SPMI. In Dolj, all ten physicians improved on MISI and SPMI, whereas eight improved, one was unchanged, and one decreased on MIBI.

**Figure 2:**
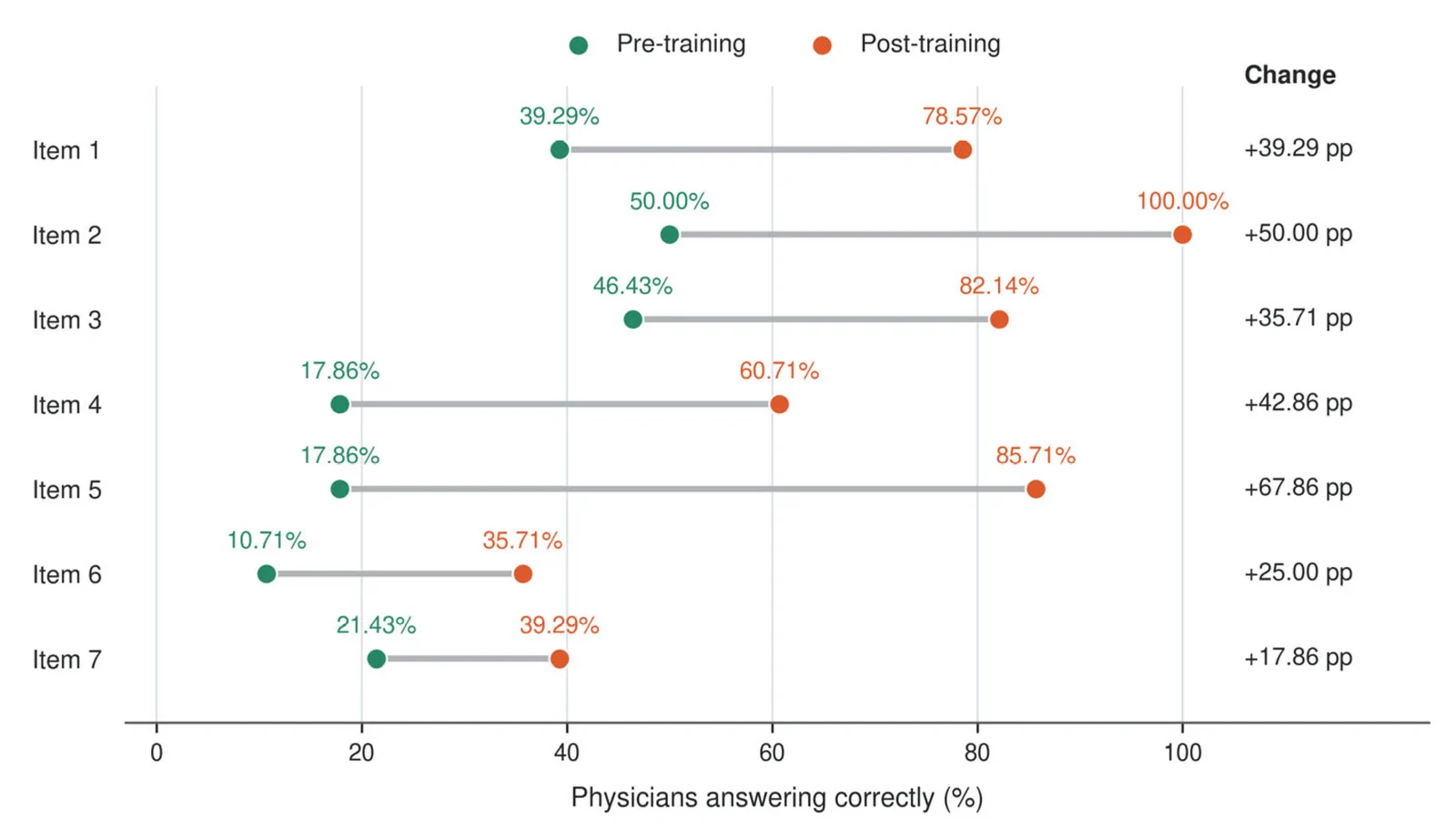
Percentage of physicians answering each MISI knowledge item correctly before and after training (*n* = 28); segments show the item-specific change in percentage points. No competence threshold was prespecified.

**Figure 3:**
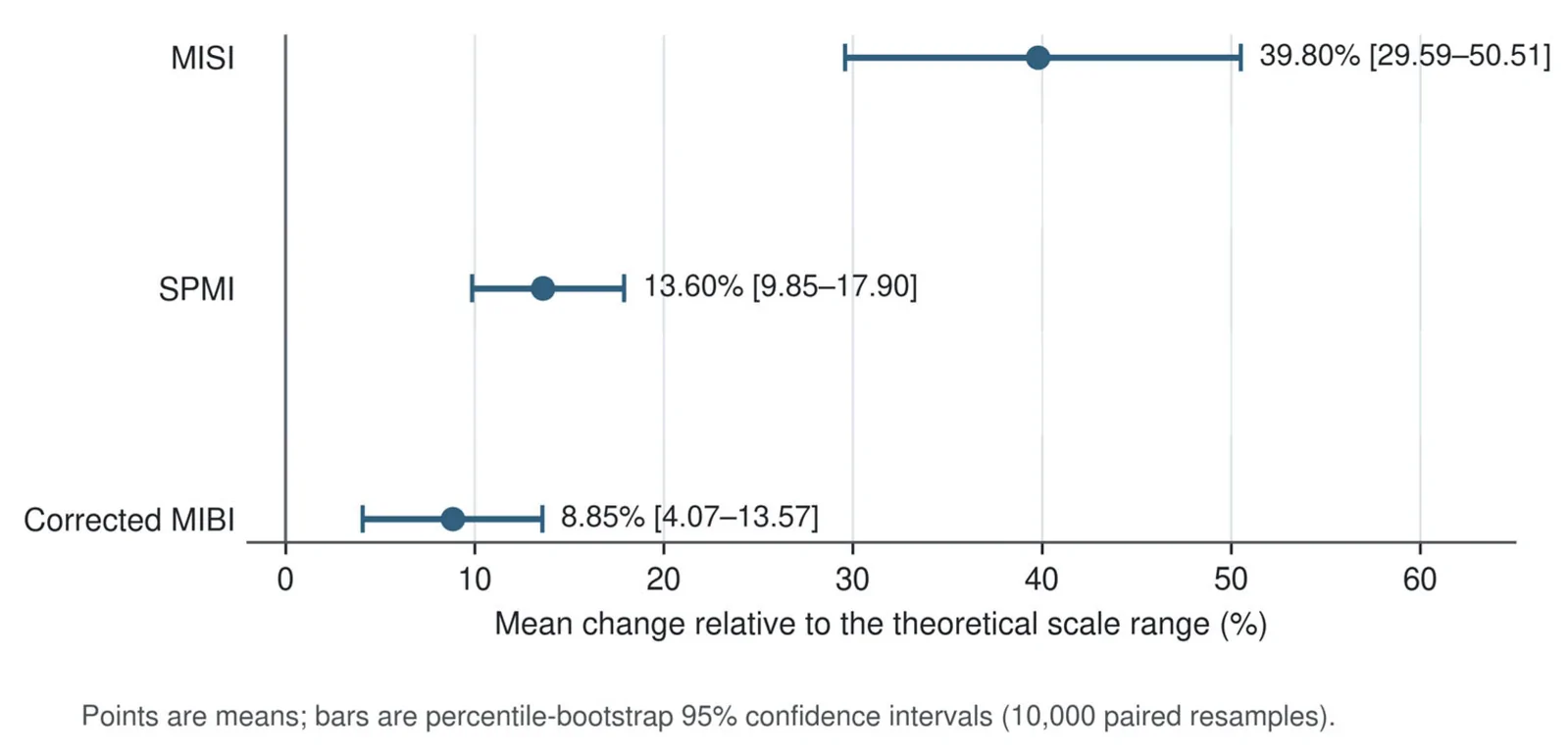
Mean pre–post changes as percentages of each instrument’s theoretical range, with percentile-bootstrap 95% confidence intervals. Cross-instrument comparisons are descriptive because response formats, reliability, and ceiling effects differ.

All seven MISI items showed descriptive increases after training (Table 2; Figure 2). The wording of the seven administered MISI items is provided in Appendix A (Table A1). The largest absolute increase occurred for item 5, from 17.86% to 85.71% correct (+67.86 percentage points). Exact McNemar tests provided nominal evidence of change for items 1–6; after Holm adjustment across seven comparisons, items 1–5 remained statistically supported, whereas the changes in items 6 and 7 were inconclusive. Items 6 and 7 also remained the least frequently answered correctly after training, at 35.71% and 39.29%, respectively. These percentages were interpreted descriptively because no competence threshold had been prespecified. The corresponding normalised scale-level changes are shown in Figure 3.

The two MI-inconsistent MIBI items were examined separately on their original response scales, on which lower post-training values represented change in the MI-consistent direction (Table 3). Item 5 decreased by a mean of 0.54 points; 13 physicians reported lower values, ten were unchanged, and five reported higher values (raw *p* = 0.051; Holm-adjusted *p* = 0.103). Item 9 decreased by a mean of 0.25 points, with eight lower, 11 unchanged, and nine higher post-training responses (raw and adjusted *p* = 0.374). Thus, both items changed descriptively in the expected direction, but neither item-level comparison was statistically conclusive.

The exploratory MIBI domain analysis showed that the mean of the ten MI-aligned items increased from 4.69 ± 0.71 to 5.14 ± 0.51, corresponding to a mean paired increase of 0.45 points (bootstrap 95% CI: 0.21–0.68) and r_rb_ = 0.81 (95% CI: 0.48–1.00; Holm-adjusted *p* < 0.001). Twenty-three physicians increased, two were unchanged, and three decreased on this domain. By contrast, the mean of the two MI-inconsistent items, retained on their original scales, decreased from 4.54 ± 1.17 to 4.14 ± 1.16 (mean paired change −0.39; 95% CI: −0.84–0.07). Fourteen physicians reported lower values, six were unchanged, and eight reported higher values; the effect in the favourable direction was r_rb_ = 0.40 (95% CI: −0.06–0.78; Holm-adjusted *p* = 0.100). Thus, the corrected MIBI increase was accompanied by a clear increase in the MI-aligned domain, whereas the combined change in the two MI-inconsistent items remained inconclusive.

The seven-item MISI composite showed limited internal consistency before training (KR-20 = 0.42). After training, all 28 physicians answered item 2 correctly, so this invariant item was excluded and the resulting six-item KR-20 was 0.63. Because the pre- and post-training coefficients were calculated from different item sets, their difference was not interpreted as evidence that reliability improved following training. Accordingly, the scale-level MISI change was not treated as a precise estimate of a single unidimensional construct. Interpretive priority was given to the seven item-specific changes, including the persistence of low post-training performance on items 6 and 7. Cronbach’s alpha for corrected MIBI was 0.80 before and 0.71 after training, based on 26 and 28 complete responses, respectively. For SPMI, Cronbach’s alpha was 0.86 before and 0.82 after training, based on 27 and 28 complete responses, respectively. Given the small sample and limited number of items, all reliability coefficients were treated as descriptive estimates.

All six SPMI items increased, and each item-level change remained statistically supported after Holm adjustment across the six comparisons (all adjusted *p* ≤ 0.005). The largest mean increases concerned perceived preparedness to conduct a vaccination consultation in the spirit of MI (+1.68 points), ease of discussing vaccination with a hesitant patient (+1.43), and comfort with empathetic listening (+1.39). These findings indicate that the increase in self-perceived ability was distributed across the assessed components rather than being attributable to a single item.

Complete-case sensitivity analyses supported the robustness of the main findings. For corrected MIBI, the complete-case analysis included 26 physicians and showed an increase from 4.29 to 4.79 (r_rb_ = 0.85; *p* < 0.001), compared with r_rb_ = 0.75 in the available-item analysis. For SPMI, the complete-case analysis included 27 physicians and yielded mean scores of 7.88 and 9.06 before and after training, respectively (r_rb_ = 0.99; *p* < 0.001). Thus, the direction and statistical interpretation of the main findings were unchanged after restricting the analyses to physicians with complete item-level data.

In the immediate course evaluation, all 28 participants selected the highest response category (5/5) for perceived learning, anticipated influence on practice, organisation, scientific content, and lecturer quality. The ratings indicate uniformly favourable immediate acceptability but had no between-participant variation and therefore offered limited discrimination. Anticipated influence on practice was interpreted as a perception, not as evidence of subsequent behavioural change.

### 3.3. Exploratory Patient Composite Change and Physician–Patient Associations

Patient records were available for 6 of the 18 physicians from Iași and 9 of the 10 physicians from Dolj; the remaining physicians had no patient records in the supplied source files rather than failed identifier matches. The linked component comprised 61 patients in Iași and 90 in Dolj, yielding 151 patient records nested within 15 physicians. The physician remained the independent unit of analysis. Across these 15 physicians, the mean patient composite score increased from 4.47 ± 0.88 before consultation to 5.02 ± 0.70 after consultation. The mean physician-level patient composite change was 0.55 ± 0.37 points (percentile-bootstrap 95% CI: 0.38–0.75), and the median change was 0.45 [IQR: 0.33–0.74]. All 15 physician-level mean changes were positive (r_rb_ = 1.00; *p* < 0.001). The corresponding mean changes were 0.62 points in Iași (95% CI: 0.39–0.84) and 0.50 points in Dolj (95% CI: 0.30–0.80). Weighting physician-level means by the number of contributing patients produced a pooled estimate of 0.55 points, identical to the unweighted estimate to two decimal places. Because participation in this linked component covered only 15 physician clusters and was not accompanied by a comparison group, these results are retained as exploratory evidence about a proximal patient response, not as an estimate of clinical effectiveness or vaccination uptake.

No consistent association was observed between mean patient composite change and physician-level changes in MISI, corrected MIBI, or SPMI across the two centres (Table 4). In Iași, the largest observed correlation involved MISI change (rho = 0.74; bootstrap 95% CI: −0.30–1.00; raw *p* = 0.096), but the interval was very wide, and the result did not remain statistically supported after Holm adjustment across six correlations (adjusted *p* = 0.574). In Dolj, the corresponding MISI estimate was close to zero (rho = −0.05; 95% CI: −0.88–0.78; raw *p* = 0.903). The confidence intervals for all six correlations included zero and spanned both potentially negative and positive associations. These correlation analyses were considered hypothesis-generating because the effective centre-specific samples comprised only 6 and 9 physicians.

## 4. Discussion

The present study identified higher immediate post-training scores across three complementary physician-level domains: MI knowledge, self-reported MI-aligned communication behaviour, and self-perceived ability to provide vaccination counselling. The paired changes were accompanied by large rank-biserial estimates; however, these estimates describe within-participant score shifts in an uncontrolled pre–post design and should not be interpreted as causal effects of the training programme. The largest normalised change was observed for MI knowledge. This cross-instrument comparison remains descriptive because the three measures differed in response format, reliability, and susceptibility to ceiling effects. The low baseline MISI scores and the persistence of weaknesses in specific knowledge items nevertheless indicate areas in which further professional education may be warranted.

### 4.1. Changes in Self-Reported MI-Aligned Communication

The corrected composite MIBI score increased after training, supporting an improvement in self-reported MI-aligned communication. The two MI-inconsistent items—immediately trying to convince the patient and telling the patient what the physician would do in their place—also decreased descriptively. However, these two item-level changes were not individually conclusive, and the data therefore support a directionally consistent pattern rather than proving genuine adoption of the spirit of MI. The concurrent increases in knowledge, behaviour, and perceived self-efficacy are nevertheless coherent with observations from studies of empathetic communication in vaccinology [22,23].

### 4.2. Interpretation in Relation to Previous Training Studies

The pattern of change is informative precisely because it was uneven. Knowledge changed more than self-reported behaviour or confidence after normalisation to the theoretical scale ranges. This may reflect low baseline knowledge and the relative ease with which declarative content changes during a single session, whilst communication habits require rehearsal, feedback, and use in clinical encounters. Immediate assessment may also favour recall and confidence outcomes before behavioural integration can occur; ceiling effects were more plausible for MIBI and SPMI than for MISI. These explanations are hypotheses rather than mechanisms demonstrated by the present data: immediate self-reported assessment cannot establish a temporal sequence from knowledge or confidence to observable clinical practice. Similar pre–post gains across knowledge, perceived application, or self-efficacy have been reported after MI-related vaccination training in Portugal and amongst general-practice trainees, whereas programmes using observed standardised-patient encounters show that some relational competencies improve without all domains reaching proficiency [28,29,30]. Curriculum-development and implementation studies likewise emphasise practice, refresher sessions, and fidelity assessment rather than one-off knowledge transfer [31]. The large MISI rank-biserial estimate must nevertheless be interpreted alongside the weak pre-training internal consistency of the seven-item composite. It indicates a highly consistent upward ordering of paired scores, not a highly precise estimate of a single latent competency. The item-level results are therefore more diagnostic: five items remained supported after multiplicity adjustment, whereas items 6 and 7—both requiring application of MI-consistent responses to concrete patient scenarios—remained below 40% correct. Their relative difficulty suggests that recognising principles may develop before selecting an MI-consistent response in a simulated consultation. This distinction also helps explain why the two MI-inconsistent behaviour items did not change conclusively. Directive habits such as immediately attempting to persuade or offering one’s own preferred choice may be resistant to a brief intervention and may require observed practice, tailored feedback, and reinforcement.

### 4.3. Link to Patient-Level Effects

The linked physician–patient component showed a favourable mean patient composite change after vaccination counselling, with positive physician-level mean changes observed for all 15 physicians contributing patient records. This finding provides patient-level evidence of change following consultations delivered by trained physicians, but it should not be interpreted as a causal effect of the training programme because no comparison group of patients counselled by untrained physicians was available in the present analysis. Moreover, larger changes in physician MISI, corrected MIBI, or SPMI scores were not consistently associated with larger mean patient changes. The largest observed correlation, involving MISI in Iași, was imprecise and was not statistically supported after multiplicity adjustment. The proposed pathway linking physician training, physician competency change, and patient outcomes therefore remains plausible but was not demonstrated by the exploratory correlation analyses. Establishing such a pathway would require a larger, prospectively designed study with an appropriate comparison group and sufficient numbers of physician clusters. The absence of a clear competency–patient correlation should not be interpreted as evidence that physician competency is irrelevant. With only 6 and 9 physician clusters by centre, restricted variation in some post-training scores, measurement error in the low-reliability MISI composite, and heterogeneous patient-level determinants, the analysis had little capacity to identify a stable cross-level association. It instead shows that the proposed pathway was not demonstrated by these data.

### 4.4. Implications

The findings support a staged rather than one-off approach to MI-based continuing medical education. Initial teaching can address core knowledge and introduce the relational logic of MI, but the residual item-level weaknesses and inconclusive change in MI-inconsistent behaviours argue for subsequent rehearsal, observed or recorded consultations, structured feedback, and refresher sessions. Future programmes should prespecify educationally meaningful competency criteria, document delivery fidelity at each site, and separate knowledge recall, observed communication behaviour, self-efficacy, and patient outcomes. Longer-term assessment is required to determine whether immediate gains persist and transfer to routine practice. In undergraduate and residency curricula, repeated exposure may be more defensible than a single isolated module. These priorities are particularly relevant in Central and Eastern European settings, where vaccine hesitancy and changing vaccination policies place increasing demands on primary-care communication [13].

### 4.5. Limitations

The study has several limitations. (1) The pre–post design without a randomised control group does not exclude testing, learning, regression-to-the-mean, or social-desirability effects. (2) Communication behaviour and perceived ability were self-reported rather than observed in practice. (3) Assessment occurred immediately after training, so retention and transfer to routine consultations were not measured. (4) The voluntary, self-selected non-probability sample was small, particularly for centre-specific analyses, and age, sex, years in practice, practice setting, and previous communication training were unavailable. The sample cannot therefore be adequately contextualised, selection bias cannot be characterised, and possible effect heterogeneity across these factors cannot be examined. (5) Although both sessions followed a common programme framework, no formal fidelity checklist, recording, or independent observation was used; equivalent delivery in Iași and Craiova cannot be established. (6) Patient records were available for only 15 of the 28 physicians. Although 151 patients contributed, observations were clustered within 15 practices, leaving a small effective sample; without a comparison group, patient composite change cannot be attributed to the training. (7) A shortened Romanian-language MISI was used. The pre-training seven-item KR-20 was 0.42, whereas the post-training coefficient of 0.63 was calculated after excluding an invariant item. These coefficients are not directly comparable and their difference does not demonstrate improved reliability; the composite is not a precise estimate of a single knowledge construct, and item-level findings deserve greater interpretive weight. (8) The exploratory item-level and physician–patient analyses were not powered for definitive inference. Holm adjustment reduced false-positive risk within specified comparison families but cannot remedy the imprecision of small samples. Larger controlled studies with observed consultations, fidelity assessment, relevant physician covariates, and longer follow-up are required.

## 5. Conclusions

Immediate post-training scores were higher across physicians’ motivational interviewing knowledge, self-reported MI-aligned communication behaviour, and self-perceived ability to provide vaccination counselling. The linked physician–patient component also showed a favourable physician-aggregated mean patient composite change following consultation. However, physician competency changes were not consistently associated with patient change, and the uncontrolled design precludes attributing either finding specifically to the training programme. Taken together, these results suggest that structured MI-based training warrants further evaluation as a potential component of continuing medical education for family physicians involved in vaccination counselling. Larger controlled studies incorporating observed consultations, sufficient numbers of physician clusters, and longer-term follow-up are required to determine whether the observed immediate changes translate into sustained professional competencies and patient benefit.

## Figures and Tables

**Table 1:** Pre–post changes in physicians’ motivational interviewing outcomes by centre and in the pooled sample.

Outcome	Centre (*n*)	Pre Mean ± SD	Post Mean ± SD	Mean Paired Change (95% CI)	Wilcoxon *p*	r_rb_
MISI, % correct	Iași (18)	27.78 ± 21.06	69.84 ± 26.34	+42.06	<0.001	0.96
	Dolj (10)	31.43 ± 21.08	67.14 ± 16.56	+35.71	0.005	1.00
	Pooled (28)	29.08 ± 20.75	68.88 ± 23.02	+39.80 (29.59–50.51)	<0.001	0.98
Corrected MIBI	Iași (18)	4.37 ± 0.74	4.95 ± 0.36	+0.58	0.003	0.82
	Dolj (10)	4.23 ± 0.39	4.42 ± 0.61	+0.19	0.109	0.60
	Pooled (28)	4.32 ± 0.63	4.76 ± 0.52	+0.44 (0.20–0.68)	<0.001	0.75
SPMI	Iași (18)	8.27 ± 1.06	9.32 ± 0.51	+1.06	<0.001	0.98
	Dolj (10)	7.04 ± 1.47	8.57 ± 1.05	+1.53	0.005	1.00
	Pooled (28)	7.83 ± 1.33	9.05 ± 0.82	+1.22 (0.89–1.61)	<0.001	0.99

Note: Values are mean ± SD. Mean paired changes are presented with bootstrap 95% confidence intervals for the pooled main outcomes, which were the prespecified inferential focus; centre-specific estimates are exploratory and are shown without confidence intervals because the small subgroup sizes would yield unstable interval estimates; median paired changes and bootstrap 95% confidence intervals for the pooled r_rb_ estimates are reported in the accompanying text. Within-centre *p* values are exploratory and unadjusted; pooled *p* values are Holm-adjusted across the three main scale-level outcomes. CI, confidence interval; r_rb_, matched-pairs rank-biserial correlation; SD, standard deviation.

**Table 2:** Item-level changes in motivational interviewing knowledge (MISI) in the pooled sample (*n* = 28).

MISI Item	Pre *n*/N (%)	Post *n*/N (%)	Δ, pp	Discordant 0→1/1→0	Exact *p*	Holm *p*
Behaviour-change determinants	11/28 (39.29%)	22/28 (78.57%)	+39.29	14/3	0.013	0.038
Core MI skills	14/28 (50.00%)	28/28 (100.00%)	+50.00	14/0	<0.001	<0.001
MI-consistent counselling attitude	13/28 (46.43%)	23/28 (82.14%)	+35.71	10/0	0.002	0.009
Response to multiple-vaccine concern	5/28 (17.86%)	17/28 (60.71%)	+42.86	13/1	0.002	0.009
MI spirit attitudes	5/28 (17.86%)	24/28 (85.71%)	+67.86	19/0	<0.001	<0.001
Response to pneumococcal-vaccine question	3/28 (10.71%)	10/28 (35.71%)	+25.00	8/1	0.039	0.078
Simulated hesitant-patient consultation	6/28 (21.43%)	11/28 (39.29%)	+17.86	7/2	0.180	0.180

Note: Discordant pairs are presented as the number changing from incorrect to correct (0→1) and from correct to incorrect (1→0). Raw exact two-sided McNemar *p* values and Holm-adjusted *p* values across seven comparisons are shown. Δ, change in percentage points; N, number of physicians with a valid paired response.

**Table 3:** Pre–post changes in the two MI-inconsistent MIBI items in the pooled sample (*n* = 28), reported on their original scales.

MIBI Item	Pre-Training	Post-Training	Mean Change	Raw *p*	Holm *p*
Item 5	4.29 ± 1.18; 4.50 [4.00–5.00]	3.75 ± 1.62; 4.00 [2.00–5.00]	−0.54	0.051	0.103
Item 9	4.79 ± 1.47; 5.00 [4.00–6.00]	4.54 ± 1.69; 5.00 [3.00–6.00]	−0.25	0.374	0.374

Note: Values are mean ± SD and median [interquartile range]. Lower post-training values indicate change in the MI-consistent direction. Raw two-sided Wilcoxon *p* values and Holm-adjusted *p* values across the two comparisons are shown. MIBI, Motivational Interviewing Behaviour Instrument; SD, standard deviation.

**Table 4:** Exploratory associations between physician-level score changes and mean patient composite change.

Centre	Physician Outcome	Physicians	Patients	Spearman rho (95% CI)	Raw *p*	Holm *p*
Iași	MISI change	6	61	0.74 (−0.30–1.00)	0.096	0.574
	MIBI change	6	61	0.03 (−1.00–1.00)	0.957	1.000
	SPMI change	6	61	0.09 (−1.00–1.00)	0.872	1.000
Dolj	MISI change	9	90	−0.05 (−0.88–0.78)	0.903	1.000
	MIBI change	9	90	0.17 (−0.73–0.79)	0.656	1.000
	SPMI change	9	90	0.28 (−0.63–0.90)	0.472	1.000

Note: Spearman rho values are presented with percentile-bootstrap 95% confidence intervals based on 10,000 physician-level resamples. Raw two-sided *p* values and Holm-adjusted *p* values across six correlations are shown. CI, confidence interval; MIBI, Motivational Interviewing Behaviour Instrument; MISI, Motivational Interviewing Skills in Immunisation knowledge component; SPMI, self-perceived motivational interviewing ability.

## Data Availability

The anonymised data are available from the corresponding author upon reasonable request.

## Abbreviations

The following abbreviations are used in this manuscript:

CI	Confidence interval
CME	Continuing medical education
CPSS	Centre for Health Policies and Services
KR-20	Kuder–Richardson Formula 20
MIBI	Motivational Interviewing Behaviour Instrument
MI	Motivational interviewing
MISI	Motivational Interviewing Skills in Immunisation
NIP	National Immunisation Program
SNMF	National Society of Family Medicine in Romania
SPMI	Self-perceived motivational interviewing ability
VH	Vaccine hesitancy
WHO	World Health Organisation

## Appendix A

Table A1 identifies the seven MI knowledge items administered to physicians and presents the proportion answered correctly before and after training in the pooled sample (*n* = 28). Item descriptions are concise English renderings of the Romanian-language form used in the study.

**Table A1:** Motivational interviewing knowledge items actually administered and percentage correct before and after training.

Item	Item Statement (Knowledge Component)	% Correct (pre → post)
Item 1	Three determinants of personal behaviour change according to MI	39.29% → 78.57%
Item 2	Core skills used in motivational interviewing	50.00% → 100.00%
Item 3	MI-consistent attitude when counselling a hesitant person	46.43% → 82.14%
Item 4	Best MI-consistent response to concern about receiving multiple vaccines	17.86% → 60.71%
Item 5	Four fundamental attitudes expressing the spirit of MI	17.86% → 85.71%
Item 6	Best MI-consistent response to a patient questioning pneumococcal vaccination	10.71% → 35.71%
Item 7	MI-consistent responses in a simulated consultation with a vaccine-hesitant older adult	21.43% → 39.29%

Note: Items 6 and 7 remained below 40% correct after training. This is a descriptive observation, not a prespecified competence threshold.
